# Agreement between maximum and mean handgrip strength measurements in cancer patients

**DOI:** 10.1371/journal.pone.0270631

**Published:** 2022-07-01

**Authors:** Rayne de Almeida Marques, Vanusa Felício de Souza, Thainá Cezini do Rosario, Maria Rita Pereira da Silva Garcia, Taísa Sabrina Silva Pereira, José Luiz Marques-Rocha, Valdete Regina Guandalini

**Affiliations:** 1 Postgraduate Program in Nutrition and Health, Health Sciences Center, Federal University of Espírito Santo, Vitória, Espírito Santo, Brazil; 2 Department of Integrated Health Education, Health Sciences Center, Federal University of Espírito Santo, Vitória, Espírito Santo, Brazil; 3 Department of Health Sciences, Universidad de las Américas Puebla, Cholula, Puebla, México; Universita degli Studi di Milano, ITALY

## Abstract

**Background and aim:**

Handgrip strength (HGS) can be used to identify probable sarcopenia, by measuring maximum strength and/or through the average of three measurements. This study analyzed the agreement between maximum and mean HGS measurements in identifying probable sarcopenia in cancer patients.

**Methods:**

Adult individuals of both sexes diagnosed with malignant neoplasm were evaluated. HGS (kg/f) was measured in both hands and nutritional status defined by the Patient-Generated Subjective Global Assessment (PG-SGA). Bland-Altman, Intraclass Correlation Coefficient (ICC), and Cronbach’s Alpha tests were applied to assess the agreement between measurements.

**Results:**

One hundred forty-one patients aged 60.0 ± 14.2 years were evaluated. There was a predominance of elderly (57.4%), male (53.2%), and non-white (58.2%) individuals, with tumors located in the lower gastrointestinal tract (GIT) (36.9%) and with suspected or some degree of malnutrition (61.0%). For men, the Bland-Altman test showed a mean error of 1.37 (95% CI—1.03 to 3.80) for dominant HGS (DHGS) and 1.50 (95% CI—1.60 to 4.60) for non-dominant HGS (NDHGS), while for women the values were 1.34 (95% CI—0.27 to 2.95) and 1.14 (95% CI—1.10 to 3.39), respectively. The ICC showed excellent reproducibility (> 0.90) and the Cronbach’s Alpha was satisfactory (0.99).

**Conclusion:**

Despite the satisfactory agreement observed between maximum and mean HGS values, in this study, individuals of both sexes with probable sarcopenia were better identified through mean values.

## Introduction

Cancer patients are exposed to several specific and non-specific factors that cause muscle mass dysfunction, favoring protein catabolism [1]. Among these factors, we can mention antineoplastic treatment, factors derived from the tumor, age, the presence of comorbidities, malnutrition, and physical inactivity [1].

The low amount of muscle, accompanied by decreased muscle strength and physical performance, characterize sarcopenia, which is classified as primary when caused by aging, and secondary, when it results from diseases such as cancer [2, 3]. Oflazoglu et al. [4] evaluated the presence of sarcopenia in newly diagnosed cancer patients and found a prevalence of 16.7%, which was higher in older males with lower body mass index (BMI) and poor physical performance.

Another study that investigated sarcopenia and its predictive value for postoperative complications in patients with gastric cancer found that 14.4% of the 507 patients evaluated were sarcopenic [5]. Furthermore, it was noted that the reduction in muscle mass and the low handgrip strength (HGS) in this group mediated the adverse impacts of sarcopenia on postoperative complications [5].

Muscle strength is one of the phenotypes used in the diagnosis of sarcopenia [2]. The European Working Group on Sarcopenia in Older People 2 (EWGSOP2), responsible for the European Consensus on Sarcopenia, recommended, in its latest version, the measurement of HGS to measure muscle strength, as it is a reliable measure associated with loss of mass and low level of muscle strength [2, 6]. When reduced, HGS indicates probable sarcopenia [2].

The HGS test is an objective, fast, easy, and low-cost method that has been used in hospital practice, specialized clinical settings, and in collective health services [2]. In cancer patients, reduced HGS values are associated with post-operative complications, length of stay, chemotherapy toxicity, functional status, short- and medium-term survival, and cost to the health system [7, 8]. However, there are two methods for obtaining HGS, which makes it difficult to standardize the procedure to be adopted in clinical practice and, consequently, to compare results. The most referenced HSG protocol is the one recommended by the American Society of Hand Therapists (ASHT), in which both the average of three measurements (mean HGS) and the highest value of two or three measurements (maximum HGS) can be used [9]. There are few studies that seek to clarify whether there are differences between these two ways of determining HGS. Most deal with small samples, which compromises the reliability of the results [10, 11].

Thus, investigating the difference in the use of mean or maximum HGS values contributes to the standardization and reliability of the method for future research and clinical practice. In this sense, the aim of this study was to analyze the agreement between maximum and average HGS values in the identification of probable sarcopenia in cancer patients according to sex.

## Materials and methods

### Subjects and study period

This cross-sectional study with a non-probabilistic convenience sample was carried out from 2017 to 2019, in a public hospital located in the city of Vitória-ES, Brazil. The study included individuals aged 20 years or over, of both sexes, with a confirmed diagnosis of solid tumors determined by the International Classification of Diseases for Oncology (ICD-O) [12], regardless of type and anatomical location, admitted to the General and Reparatory Surgery Unit for surgical treatment, and who had nutritional status assessment and HGS measurement performed within the first 48 hours of hospital admission. Patients with cognitive and neurological alterations reported in medical records, in respiratory isolation, under palliative care, and those who were unable to perform any of the steps of the applied protocol were excluded.

### Data collection and study variables

Data collection took place through interviews conducted by the researchers responsible for the study in the pre-surgical period, using a specific protocol. To minimize possible sample selection and data collection biases, all researchers were properly trained to apply the instruments and measure HGS. Hospitalizations and surgical indications were monitored daily so that all individuals could be evaluated during the study period.

The sociodemographic variables age, sex, and self-reported race/color were collected and later categorized into adult (< 60 years of age) and elderly (≥ 60 years of age) [13], male and female, and white and non-white, respectively. Location of the tumor was obtained from medical records and grouped into upper gastrointestinal (GIT), lower GIT, adnexal glands (liver, pancreas, and biliary tract), and others for the other locations found (lung, unknown behavior, ovary, cervical, mediastinum, peritoneum, connective tissue, and thyroid). Tumor location was classified as such due to predominance in the GIT, justified by the fact that the hospital where this study was carried out is a reference for GIT surgeries. After this stage, participants were submitted to HGS measurement and to assessment of nutritional status by Patient-Generated Subjective Global Assessment (PG-SGA).

### Handgrip strength (HGS)

To assess HGS we used the Jamar^®^ hydraulic handheld dynamometer [2]. HGS (kg/f) was measured according to the method recommended by the American Association for Hand Therapy (ASHT) [9]. At the time of measurement, the patient was seated, with the spine erect, knees flexed at 90°, shoulder positioned in abduction, forearm supported on the trunk, and elbow flexed at 90° [9]. The procedure was performed three times in the dominant hand (DHGS) and three times in the non-dominant hand (NDHGS), with maximum effort for about 5 seconds and a 1-min interval between measurements [9]. The value obtained from the average of the three measurements was considered as mean HGS, and the highest value of the three measurements was considered as maximum HGS. The cutoff points defined by the European Consensus on Sarcopenia were considered: < 16.0 kg/f for women and < 27.0 kg/f for men [2]. All patients with measurements below the defined cutoff points were classified as having low HGS [2].

### Patient-Generated Subjective Global Assessment (PG-SGA)

The assessment of nutritional status was performed using the PG-SGA. Individuals were classified into three categories: well nourished (A), suspected or moderately malnourished (B), and/or severely malnourished (C) [14]. For this study, the translated and validated version into Portuguese by Gonzalez et al. [14] was used, with permission to use PG-SGA/Pt-Global Platform (www.pt-global.org).

### Data analysis

Descriptive analysis expressed as means and standard deviations for continuous variables and percentages for categorical variables was performed. The Kolmogorov-Smirnov test was used to verify the normality of quantitative variables. To assess the agreement between maximum and mean HGS values of both hands, we used the Bland-Altman graphical presentation, which allows the assessment of bias, the dispersion of points around the average, and possible outliers and trends [15, 16]. The Intraclass Correlation Coefficient (ICC) was applied to assess the degree of agreement between the maximum and mean HGS. ICC was interpreted based on the Bland-Altman suggestions, and the following values were considered: unacceptable, when < 0.4; good reproducibility, from 0.41 to 0.6; very good reproducibility, from 0.61 to 0.80; and excellent reproducibility, from 0.81 to 1.0 [17]. Cronbach’s Alpha coefficient was used to assess the reliability and internal consistency of the measures evaluated. A value equal to or above 0.70 was considered satisfactory [18]. Data were analyzed using the SPSS 22.0 software and p < 0.05 was adopted for all tests. Only individuals with all data were included in the analysis, with no treatment for missing data.

### Ethical aspects

This study was approved by the Ethics and Research Committee of the Federal University of Espírito Santo, under protocol number 2.141.932/2017. Patients participated voluntarily and provided written informed consent, in accordance with the Resolutions 510/2016 and 466/12 of the National Health Council of Brazil, which regulates research with humans.

## Results

A total of 141 patients with a mean age of 60.0 ± 14.2 years were included in the study (Table 1). There was a predominance of elderly (57.4%), male (53.2%), and self-declared non-white (58.2%) individuals. The most frequent tumor location was that of the lower GIT, affecting 36.9% of those evaluated. According to the PG-SGA, 61.0% of patients had some degree of malnutrition (B + C). All demographic and clinical characteristics of the sample are shown in Table 1.

**Table 1:** Demographic and clinical characteristics of cancer patients.

Variables	n (%)
**Age** (mean ± SD)	60.0 ± 14.2
**Life stage**	**n (%)**
Adult	60 (42.6)
Elderly	81 (57.4)
**Sex**	
Male	75 (53.2)
Female	66 (46.8)
**Color**	
White	59 (41.8)
Non-white	82 (58.2)
**Tumor Location**	
Low GIT	52 (36.9)
Adnexal glands	34 (24.1)
Upper GIT	27 (19.1)
Others*	28 (19.9)
**PG-SGA**	
Well Nourished (A)	55 (39.0)
Moderate/suspected malnourished (B)	56 (39.7)
Severely malnourished (C)	30 (21.3)

PG-SGA: Patient-Generated Subjective Global Assessment; SD: standard deviation; GIT: gastrointestinal tract

*Others: lung, unknown behavior, ovary, cervical, mediastinum, peritoneum, connective tissue and thyroid.

The prevalence of low HGS in the dominant hand, when using the maximum measure as a criterion, was 29.3% (n = 22) for men and 16.7% (n = 11) for women, while for the non- dominant hand it was 36.0% (n = 27) and 19.7% (n = 13) for men and women, respectively. When considering the average of measurements as a criterion, low DHGS was observed in 32.0% (n = 24) of men and 21.2% (n = 14) of women. Regarding NDHGS, the prevalence of low strength was 37.3% (n = 28) for men and 28.8 (n = 19) for women. A significant difference was observed only in the classifications between the maximum and mean DHGS for women (p = 0.046) (Table 2).

**Table 2:** Classification of maximum and mean handgrip strength of both hands in cancer patients according to sexes.

**Men**	**DHGS max**		**p value**
**DHGS mean**	**Adequate n (%)**	**Reduced n (%)**	0.415
Adequate	34 (66.7)	17 (33.3)	
Reduced	19 (79.2)	5 (20.8)	
**Women**	**DHGS max**		**p value**
**DHGS mean**	**Adequate n (%)**	**Reduced n (%)**	0.046
Adequate	46 (88.5)	6 (11.5)	
Reduced	9 (64.3)	5 (35.7)	
**Men**	**NDHGS max**		**p value**
**NDHGS mean**	**Adequate n (%)**	**Reduced n (%)**	0.628
Adequate	29 (61.7)	18 (38.3)	
Reduced	19 (67.9)	9 (32.1)	
**Women**	**NDHGS max**		**p value**
**NDHGS mean**	**Adequate n (%)**	**Reduced n (%)**	0.172
Adequate	40 (85.1)	7 (14.9)	
Reduced	13 (68.4)	6 (31.6)	

DHGS: Dominant handgrip strength; NDHGS: Non-dominant handgrip strength; Max: Maximum.

As to the agreement assessed by ICC, excellent reproducibility was obtained (ICC > 0.90) for men and women in both hands (Table 3). The reliability and internal consistency between measurements were verified by Cronbach’s Alpha, which presented a value of 0.99, a result considered satisfactory.

**Table 3:** Agreement between maximum and mean handgrip strength in cancer patients according to sexes.

Variables (n = 141)	Mean	SD	ICC	p value	*Cronbach’s Alpha*
**Men (n = 75)**					
DHGS max (Kg/f)	33.60	11.17	0.99	<0.001	0.99
DHGS mean (Kg/f)	32.00	10.62			
NDHGS max (Kg/f)	30.81	10.94	0.99	<0.001	0.99
NDHGS mean (Kg/f)	29.33	10.38			
**Women (n = 66)**					
DHGS max (Kg/f)	21.20	5.90	0.98	<0.001	0.99
DHGS mean (Kg/f)	20.10	5.86			
NDHGS max(Kg/f)	19.43	5.31	0.99	<0.001	0.99
NDHGS mean (Kg/f)	18.27	5.16			

ICC: Intraclass Correlation Coefficient; SD: standard deviation; DHGS: Dominant handgrip strength; NDHGS: Non-dominant handgrip strength; Max: Maximum.

For triplicate measurements, the DHGS presented a coefficient of variation (CV) of 33.2% for men and 29.1% for women, while for the NDHGS, the CV was 35.4% and 25.4% for men and women, respectively (data not shown in table).

**Fig 1:**
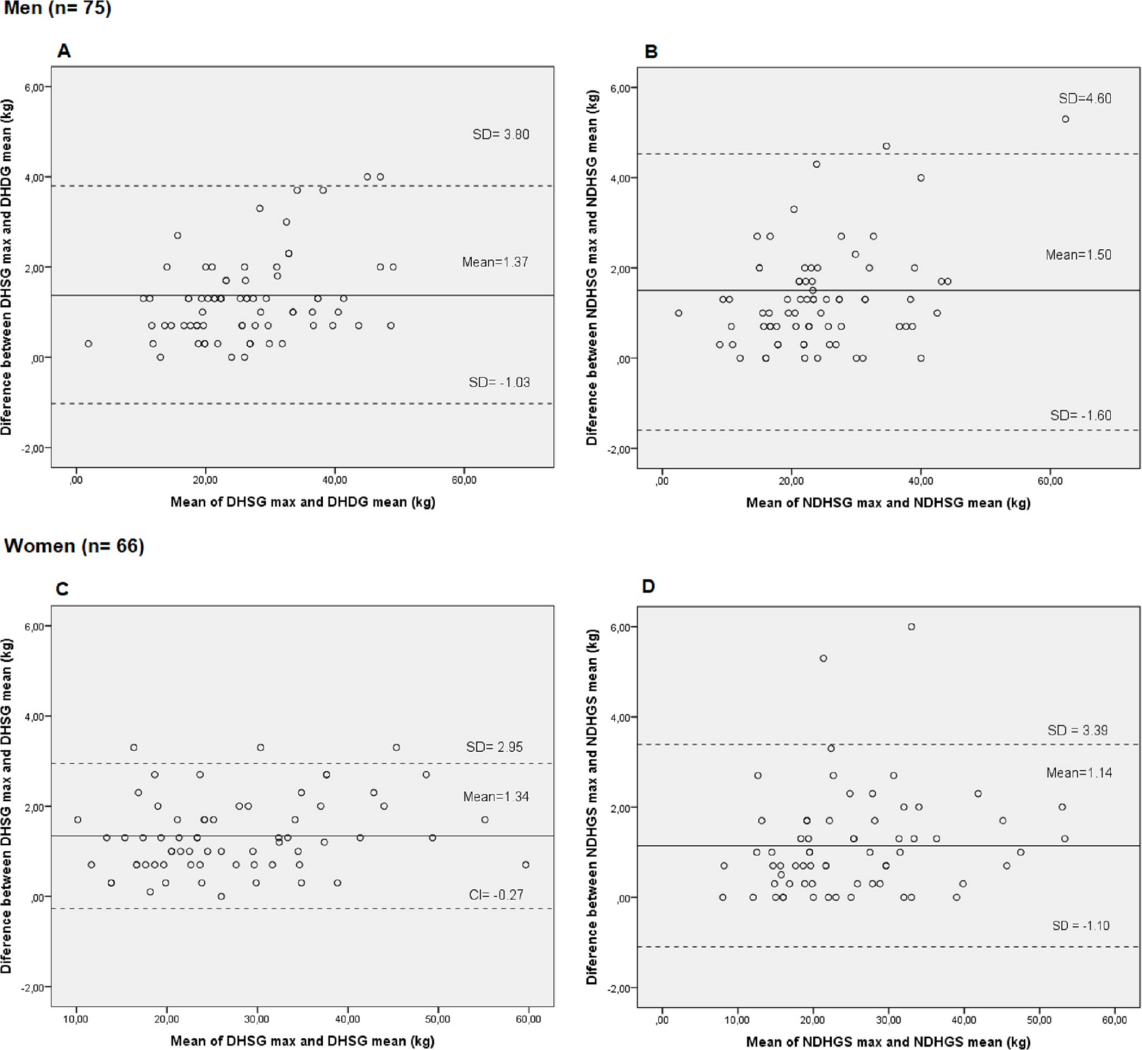
(A-D). Bland-Altman limits of agreement between the maximum handgrip strength of the dominant (DHGS max) and non-dominant (NDHGS max) hand (kg/f) and the mean handgrip strength of the dominant (DHGS mean) and non-dominant (NDHGS mean) hand (kg/f) in cancer patients according to sex.

Fig 1 shows the Bland-Altman differences comparing the consistency between the maximum and mean HGS of both hands, according to sex. The solid line shows the mean difference between the maximum and mean HGS of both hands, while the dotted lines show the 95% confidence intervals (95% CI). It is possible to observe good agreement for both hands and for both sexes (Fig 1A–1D).

Fig 1A (DHGS) shows that the mean error between measurements (estimated bias) was 1.37 (95% CI -1.03 to 3.80), while in Fig 1B (NDHGS) the mean error was 1.50 (95% CI -1.60 to 4.60) for men. Regarding women, Fig 1C (DHGS) and 1D (NDHGS) show mean error values of 1.34 (95% CI -0.27 to 2.95) and 1.14 (95% CI -1.10 to 3.39), respectively.

## Discussion

The results presented in this study demonstrate an agreement between maximum and mean HGS values in identifying probable sarcopenia in cancer patients. Previous studies have shown no pattern as to the use of these measures at the time of their diagnostic classification [19–25]. Regardless, both maximum HGS [20–22, 25] and mean HGS [21–24] present satisfactory outcomes.

It is noteworthy that the measure of maximum strength was more used in studies that assessed nutritional status and muscle performance. The results found are consistent when compared with reference methods, such as PG-SGA and appendicular skeletal muscle mass index to predict muscle mass loss [21, 22, 26].

In situations in which body homeostasis is compromised, strength is reduced before muscle mass, becoming an important predictor of clinical outcomes with a great impact on health [27, 28]. A reduction in muscle mass, concomitant with adequate muscle strength, suggests malnutrition, while a reduction in strength alone indicates dynapenia and a decrease in both predicts sarcopenia [2, 28]. In this context, identifying muscle strength becomes an important strategy for preventing complications, as it is a reliable indicator to predict not only the conditions mentioned above, but also several unfavorable clinical outcomes that can interfere with the patient’s quality of life and prognosis [2, 28].

Pereira et al. [24], when correlating the mean HGS with anthropometric variables and with the PG-SGA of 100 outpatients with cancer, pointed out that the reduced HGS was related to longer hospital stay, functional limitations, and worse quality of life, being a predictive factor to indicate nutritional vulnerability and worse prognosis in this population.

Studies with healthy and sick individuals show higher HGS values in males than in females, when both the maximum and mean HGS were used in the evaluations, as in the present study [29–34]. This difference is related to body composition and anthropometric measurements, since men have characteristics that provide a greater degree of strength, due to a greater amount of body muscle mass [35].

In previous studies carried out in cancer patients, a higher skeletal muscle mass index and a higher percentage of fat-free mass were identified in men [36, 37]. These factors, added to muscle disposition and male bone structure, contribute to the greater strength presented by these individuals [38].

In clinical practice, it is noted that men, in addition to being stronger for physiological and anatomical reasons, tend to want to display greater strength at the time of assessment, especially in the first measurement of HGS. As a result, there may be an overestimation of values and consequent impacts on the results obtained [39].

It is also observed that other variables can influence strength, such as age–as it is inversely proportional to the strength of the individual; type of work performed–for example, a manual worker when compared to one who does not exercise this activity; in addition to ethnicity and socioeconomic factors. These factors can also influence the HGS values obtained in cancer patients [40].

Table 2 shows a higher prevalence of reduced HGS when mean values were used, and that there was a significant difference between mean and maximum HGS of women. Mean HGS is more susceptible to the protocol used, variations in individual readings, number of attempts and hand dominance [41]. It is suggested that the greatest force is not obtained in the first measurement due to unfamiliarity with the use of dynamometer, but in the subsequent one, while in the last attempt there may be muscle fatigue, contributing to lower HGS values [11, 41]. In this way, the mean of the measurements generates a value lower than the maximum HGS, which contributes to these differences occurring [41].

Regarding the assessment of dominant or non-dominant HGS to assess probable sarcopenia, a systematic review showed that most studies choose to use the dominant hand and that greater strength is expected in this hand [11]. As a way of visualizing whether the non-dominant hand follows the same characteristic, and as an innovative proposal to compare methods and measures, we also consider analyzing the non-dominant hand, with the purpose of providing information for its use, in situations of impossibility in the measurement of HGS on the dominant side, such as hand, arm or forearm surgeries in the previous 60 days [9].

Considering the CV calculated for men and women, in this study, the mean proved to be the most representative measure of HGS, although high agreement was found between measurements. In men, this variation was even greater, which corroborates and justifies the previously mentioned studies [29–35]. In this context, maximum strength could fail to classify individuals with probable sarcopenia when compared with average values.

To minimize this error, a standardized protocol and consistent instructions for carrying out the test can promote measurement reliability. It is, therefore, up to the evaluator to pay attention to the technical protocol used and guide the evaluated, clarifying the method and objectives of HGS measurement [42]. Although our study revealed an excellent agreement between maximum and mean strength measurements, in both hands and sexes, the technique must be performed correctly to ensure greater safety and reliable results.

As limitations of this study, the authors highlight the possibility of interobserver variation in the assessment of HGS. Nevertheless, when performed in triplicate by trained researchers using a calibrated dynamometer, it becomes a reliable measure. Another limitation is the lack of information on tumor staging and treatment in medical records, which can influence the nutritional status and provide a more accurate nutritional diagnosis. Finally, we emphasize that the present study evaluated the presence of probable sarcopenia through the measurement of muscle strength by the HGS test, and that it does not establish the diagnosis and severity of this syndrome, since muscle quantity, muscle quality, and physical performance, parameters necessary for this assessment, were not evaluated [2].

Nevertheless, the results reaffirmed that HGS is a sensitive indicator of reduced muscle strength. We believe that serial measures can promote the implementation of individualized nutritional strategies and reduce the chances of progression to sarcopenia.

## Conclusion

Despite the satisfactory agreement observed between maximum and mean HGS values, in this study, the mean was the measure that better identified individuals with probable sarcopenia in both sexes. The variation found between triplicate measurements suggests that the maximum force must be carefully applied and interpreted when used in isolation.

## Supporting information

S1 Data (SAV)
